# Pulmonary Embolism Mortality in Brazil from 1989 to 2010: Gender and Regional Disparities

**DOI:** 10.5935/abc.20160001

**Published:** 2016-01

**Authors:** Eduardo Sahade Darze, Juliana Borges Casqueiro, Luisa Allen Ciuffo, Jessica Mendes Santos, Iuri Resedá Magalhães, Adriana Lopes Latado

**Affiliations:** 1 Hospital Cárdio Pulmonar, Salvador, BA - Brazil; 2 Universidade Federal da Bahia, Salvador, BA - Brazil

**Keywords:** Pulmonary Embolism / epidemiology, Quality of Health Care, Brazil / epidemiology, Age Factors, Regional Medical Programs

## Abstract

**Background:**

A significant variation in pulmonary embolism (PE) mortality trends have been documented around the world. We investigated the trends in mortality rate from PE in Brazil over a period of 21 years and its regional and gender differences.

**Methods:**

Using a nationwide database of death certificate information we searched for all cases with PE as the underlying cause of death between 1989 and 2010. Population data were obtained from the Brazilian Institute of Geography and Statistics (IBGE). We calculated age-, gender- and region-specific mortality rates for each year, using the 2000 Brazilian population for direct standardization.

**Results:**

Over 21 years the age-standardized mortality rate (ASMR) fell 31% from 3.04/100,000 to 2.09/100,000. In every year between 1989 and 2010, the ASMR was higher in women than in men, but both showed a significant declining trend, from 3.10/100,000 to 2.36/100,000 and from 2.94/100,000 to 1.80/100,000, respectively. Although all country regions showed a decline in their ASMR, the largest fall in death rates was concentrated in the highest income regions of the South and Southeast Brazil. The North and Northeast regions, the lowest income areas, showed a less marked fall in death rates and no distinct change in the PE mortality rate in women.

**Conclusions:**

Our study showed a reduction in the PE mortality rate over two decades in Brazil. However, significant variation in this trend was observed amongst the five country regions and between genders, pointing to possible disparities in health care access and quality in these groups.

## Introduction

Pulmonary embolism (PE) is the third most common acute cardiovascular disease after myocardial infarction and stroke, affecting approximately 1 in 1000 people per year.^1,2^ Both hospital and population-based studies, mostly from North America, have shown that the PE mortality rate has been decreasing over the past three decades. ^3-7^ However, a recent European study has demonstrated that this fall in PE mortality could only be observed in some countries, while others showed stable or increasing rates.^8^ Demographic changes and several developments in prophylaxis, diagnosis and treatment over the past two decades^9,10^ may have affected the incidence of PE, and its case fatality and mortality rates.

Unfortunately, there is a conspicuous absence of epidemiologic data in Brazil and other Latin American countries pertaining to venous thromboembolism (VTE). Additionally, despite the fact that Brazil is among the largest global economies, similarly to all developing countries, it is plagued with marked inequalities in health care access and quality.^11^ Knowledge of the trends in mortality rate from prevalent diseases, its regional disparities and sex differences is fundamental to the conception and implementation of national and global health policies.

Using a nationwide database, we investigated the trends in PE mortality rate in Brazil over a period of 21 years. We also tried to uncover possible disparities in these trends between genders and amongst the five Brazilian geographical regions.

## Methods

Mortality data was compiled from the Brazilian National Mortality Data System directly from its web pages, which are available for the public free of charge.^12^ The database provides the age-, sex-, and region-specific number of deaths based on data collected from death certificates. Based on the International Classification of Diseases (ICD) codes, we searched for all cases that had PE as the underlying cause of death between 1989 and 2010. We used the ICD ninth revision codes 415 and 673 until 1995, and, after that, the tenth revision codes I26 and O88. The study followed the Declaration of Helsinki set of principles.

National population data were obtained from the Brazilian Institute of Geography and Statistics (IBGE), which were also stratified by sex, age and the five Brazilian geographical regions: North, Northeast, West-Central, Southeast and South.^13^ For age-adjusted rates, the 2000 Brazilian population was used as standard.

We calculated age-, gender- and region-specific mortality rates for each year between 1989 and 2010. In order to analyze overall trends in mortality rates, and gender and regional differences in these trends, direct standardization was performed using the Brazilian population of the year 2000 as standard. Linear regression models were built in order to estimate mean annual changes in mortality rates during the study period, considering the year as the independent variable and the mortality rate as the dependent variable. A significance level of 5% was used for the linear regression models. All data were analyzed using SPSS for Windows, version 17 (SPSS Inc, Chicago, Illinois, USA).

## Results

### Overall trends in mortality rates

**Figure 1:**
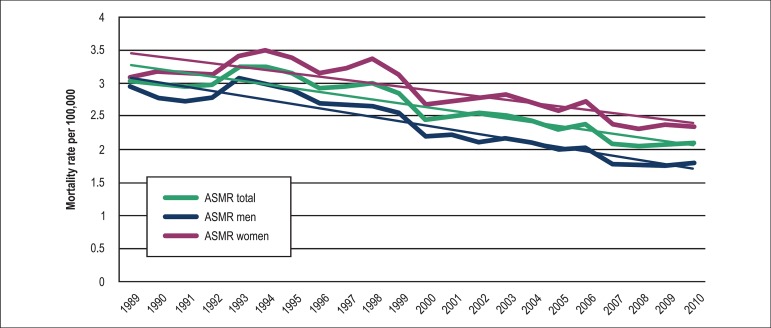
Age-standardized mortality rates (ASMR) from pulmonary embolism by gender

Between 1989 and 2010, there were 20,927,857 deaths in Brazil, of which 92,999 (51,871 women and 41,128 men) had PE listed as the underlying cause. The crude mortality rate due to PE decreased from 2.80/100,000 in 1989 to 2.62/100,000 in 2010. The age-standardized mortality rate (ASMR) also fell significantly from 3.04/100,000 to 2.09/100,000 during the same period, corresponding to a 31% drop in 21 years and a mean annual reduction of 0.057/100,000 (Figure 1).

**Figure 2:**
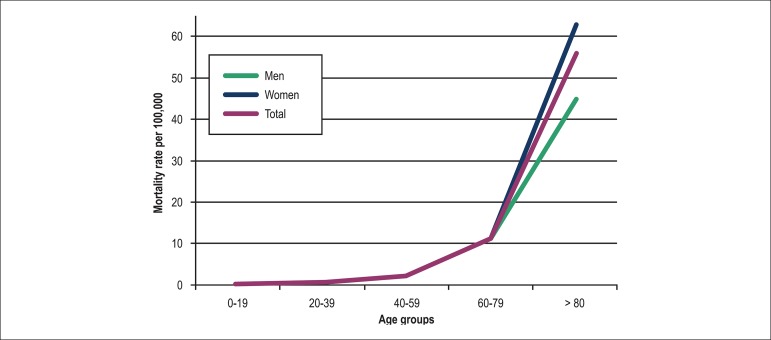
Crude mortality rates from pulmonary embolism by age group and gender for the year 2010

**Figure 3:**
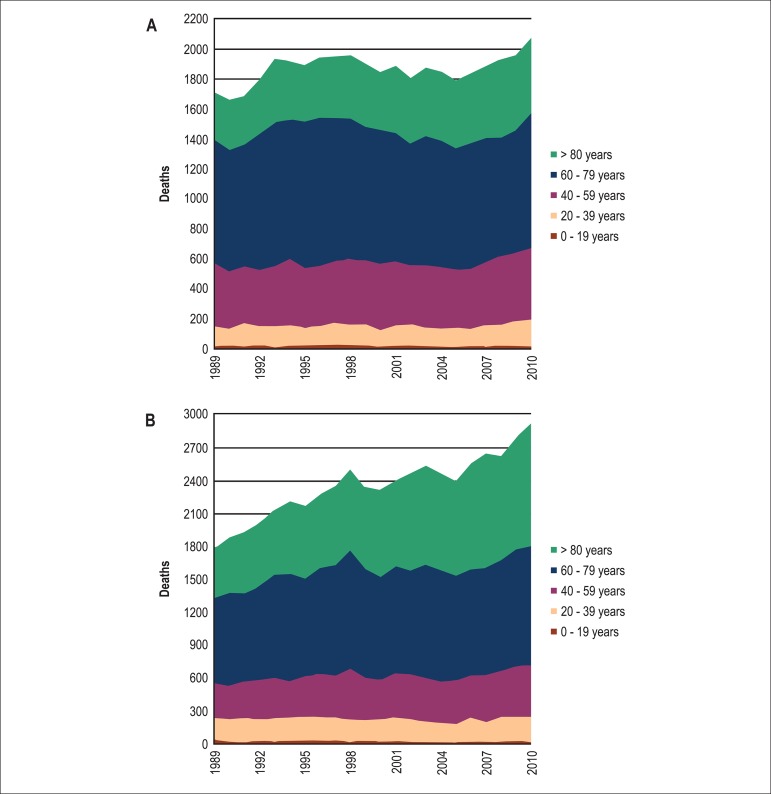
Total number of pulmonary embolism deaths by age group for men (A) and women (B)

**Figure 4:**
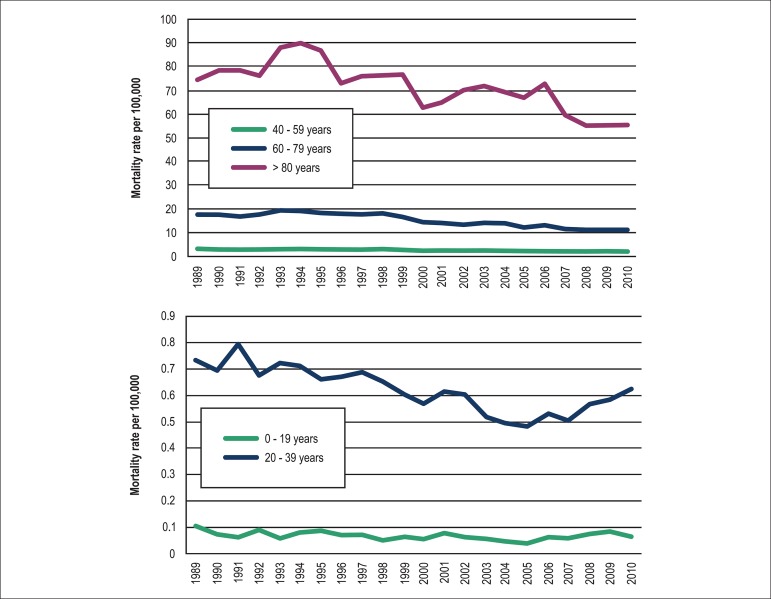
Crude mortality rates from pulmonary embolism by age group

Age-specific mortality rates due to PE varied significantly for every 20-year age stratum, increasing exponentially along the five age groups for each year analyzed. In 2010, the last year studied, the age-specific mortality rate at least tripled for each age group older than 19 years, irrespective of gender (Figure 2). The largest number of deaths occurred in the 60­79 age stratum (Figure 3). Between 1989 and 2010 the age-specific mortality rate decreased in all age groups, with the largest relative fall observed in the 60­79 age stratum (36.6%), and the lowest fall in the 20-39 age stratum (14.9%) (Figure 4).

### Trends in mortality rates and gender

**Figure 5:**
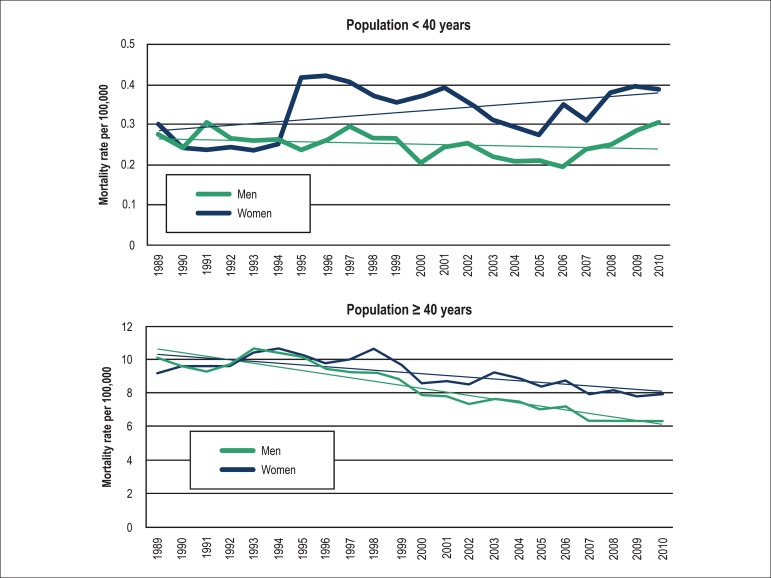
Crude mortality rates from pulmonary embolism by gender and ages < and ≥ 40 years.

Among men, the crude mortality rate and the ASMR fell along the 21-year period, from 2.45/100,000 to 2.22/100,000 and from 2.94/100,000 to 1.80/100,000, respectively. Among women, however, the crude mortality rate increased from 2.50/100,000 in 1989 to 3.01/100,000 in 2010, but the ASMR decreased from 3.10/100,000 to 2.36/100,000. Despite the significant fall in the ASMR for both sexes (men = 39%; women = 24%), in every year between 1989 and 2010, the ASMR was higher in women than in men (Figure 1). When stratified by age ≥ and < 40 years, mortality rates were similar in men and women until the mid-1990's, and from that point onwards remained consistently higher in women regardless of the age group (Figure 5).

### Trends in mortality rates and regional differences

**Figure 6:**
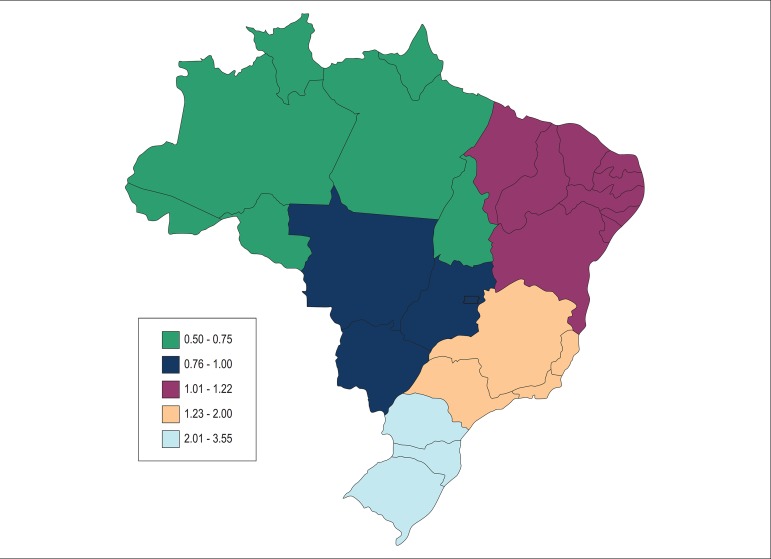
Map of age-standardized mortality rates from pulmonary embolism per 100,000 people in the five Brazilian regions - 2010

**Figure 7:**
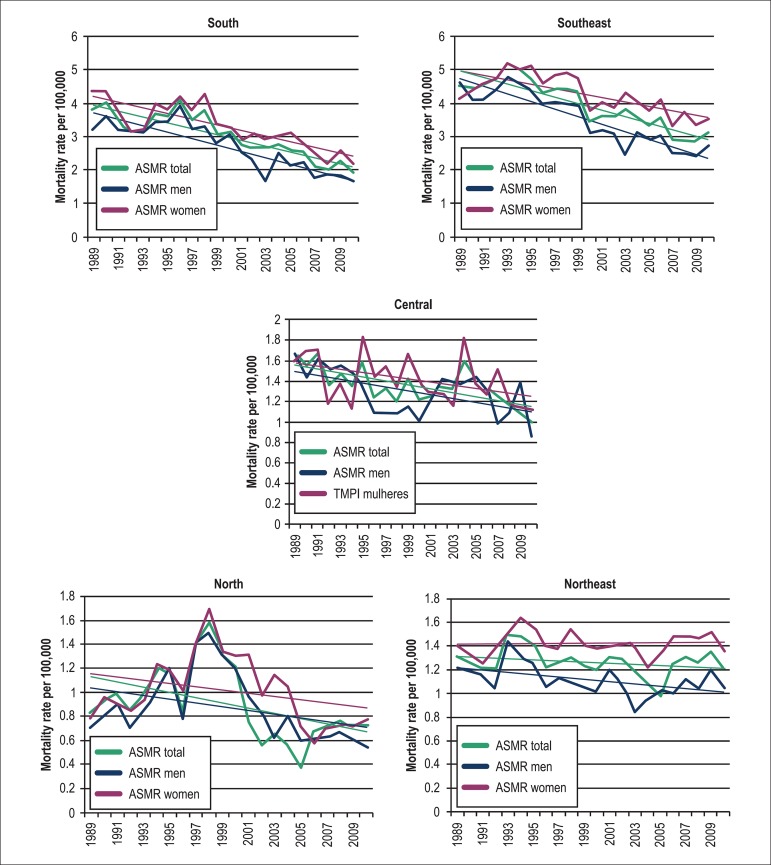
Age-standardized mortality rates (ASMR) from pulmonary embolism per geographic region and gender

In 2010, the highest ASMRs were observed in the Southeast and South regions, and the lowest rates, in the North region (Figure 6). A declining trend in the ASMR was observed in all regions, but the most marked changes were seen in the South, West-Central and Southeast regions, with drops in ASMR of 48.7%, 39.8% and 31.1%, respectively (Figure 7 and Table 1). In the North and Northeast regions, only small declines in ASMR - much below the national average - were observed along the 21-year period (12.8% and 7.2%, respectively). In the North and Northeast regions, there was no appreciable variation in the ASMR among women, and, in the Southeast and West-Central regions, the relative decrease in the ASMR between 1989 and 2010 was significantly inferior in women compared to men. Only in the South region, there were equivalent falls in ASMR between genders during the study period (men = 48.3%; women = 49.1%).

**Table 1:** Relative changes in age-standardized mortality rates from pulmonary embolism per geographic region by gender, 1989-2010

Region	Overall	Men	Women
North	-12.8%	-22.3%	-2.4%
Northeast	-7.2%	-13.3%	-2.1%
Southeast	-31.1%	-41.1%	-16.0%
South	-48.7%	-48.3%	-49.1%
West-Central	-39.8%	-47.5%	-30.7%

## Discussion

Our study showed that over the last two decades in Brazil there was a progressive and steady fall in the mortality rate from PE of approximately 31%. The same consistent decline was observed in all age groups and in both sexes, although women had a less marked fall in mortality rates along the same period. On the other hand, trends in PE mortality differed among the Brazilian regions. A steeper declining trend was observed in the South, Southeast and West­Central regions, whereas the North and Northeast regions showed a much smaller decline with no appreciable change among women. To the best of our knowledge, this is the only population-based study to investigate the long-term PE mortality trends in Latin America.

There are basically two possible explanations for the observed fall in PE mortality rates: a decrease in the incidence of VTE and/or a decrease in the case fatality rate from PE. A significant drop in the incidence of VTE seems unlikely for a few reasons. Although it had remained stable for 30 years until the 1990's, from then on several studies from different regions of the world have consistently demonstrated a progressive increase in the overall VTE incidence and its hospitalization rate.^14-18^ It is well known that the incidence of VTE increases sharply with age, particularly after the age of 60.^19^ With the aging world population, the number of VTE cases would be expected to increase. The prevalence of known risk factors for VTE has also increased during the 20-year study period. The prevalence of congestive heart failure^2^ and all types of cancer has been climbing for more than 10 years,^20^ and so has the number of surgical procedures and hospital admissions,^21^ important risk factors for venous thrombosis. Nevertheless, the relatively sharp increase in the VTE incidence observed after the 1990's seems disproportionate to the slow increments in the mean population age and prevalence of risk factors. Some authors have suggested that these observations may be attributed in part to the development and universal use of multidetector pulmonary computed tomography angiography (CTA), which has enabled a more accurate diagnosis of PE, including the detection of a more significant number of small subsegmental emboli.^14,16^

Contrary to the incidence rate, several hospital- and population-based studies have shown that, over the past 20 years, the case fatality rate of PE has dropped from around 10%-12% to 7%-8%,^22-24,14,17^ and may be the reason for the fall in the mortality rate observed in our study and several others around the world.^3-7^ Earlier and more accurate diagnosis and treatment may be the explanation for the reduction in case fatality rates.^25^ Since VTE prophylaxis in surgical and medical hospitalized patients continues to be underused^26^ and the PE incidence rises, prevention does not seem to contribute significantly to the reduction in the mortality rate. There is controversy on the actual role that the multidetector pulmonary CTA introduction in the late 1990's has played in case fatality and mortality rates. On one hand, a more accurate diagnosis would lead to earlier treatment and consequently a lower risk of death.^16,25^ However, the increase in sensitivity of the test and the detection of small and possibly insignificant subsegmental emboli may bring about a phenomenon called overdiagnosis.^14^ This is characterized by an increase in the incidence of milder or clinically insignificant forms of the disease, a consequent decline in case fatality, but no or minimal change in mortality rates, since the absolute number of deaths remains unaffected. It is not known whether this rationale applies to our findings, since there are neither parallel data on the incidence trends and case fatality rates in Brazil, nor studies documenting patterns of CTA use for the diagnosis of PE.

Our study has also shown that women had higher crude mortality rate and ASMR in every year during the study period when compared to men, even when stratified by age groups (≥ 40 years and < 40 years). The decline in mortality observed from 1989 to 2010 was also less marked in women (24% versus 39%). Gender disparities in health have been demonstrated in other cardiovascular diseases^27^ and may represent differential exposure to risks or inequalities in health care access and quality. The age-adjusted incidence of VTE appears to be slightly higher in men, with a sex ratio of 1.2:1.^1^ However, the incidence rates are somewhat higher in women during the childbearing age, reflecting the exposure to estrogen and pregnancy. On the other hand, after the age of 45, the incidence rates are generally higher in men.^1^ Regarding case fatality, most studies have shown either similar or slightly higher rates in men compared to women.^4,22,28,29^ Our observation of a higher overall ASMR in women contrasts with prior investigations from the United States, where men had higher mortality.^3,4,6^ Only one European international study showed similar findings, where women had higher age-standardized PE mortality in all countries studied, except for Poland.^8^ The reasons for the gender differences in PE mortality rates among various countries remain unexplained, but might point to sex-related variations in health care, such as proper and prompt referral for diagnostic and therapeutic procedures.^29^

Although a declining trend in ASMR was observed in all Brazilian regions, the largest fall in death rates was concentrated in the highest income regions of the South and Southeast Brazil. The North and Northeast regions, the lowest income areas, showed a less marked fall in death rates and no distinct change in PE mortality rate in women. A similar variation in region-specific PE mortality trends has also been observed when comparing different European countries.^8^ Although the majority has shown declining trends in PE mortality, some countries have shown either no change or even a clear increase in rates. If the declining PE mortality is interpreted as evidence of more accurate diagnosis and consequent earlier initiation of proper treatment, the heterogeneous mortality trends observed in the different country regions may reflect inequalities in health care delivery, with the most striking declines in mortality rates observed in the regions with the highest level of economic and social development. Nevertheless, the design of this study and its limitations should temper the strength of this association.

Uncertainties regarding the quality of physician coding of the underlying cause of death is a major concern when comparing mortality data over time and across country regions. The accuracy in assigning the cause of death may suffer a significant variation along a 20-year period and may be strikingly different in places with different levels of access to health care resources. In Brazil, only 79% of deaths were estimated to be registered, and 20% of death certificates had ill-defined codes assigned as the cause of death.^29^ Therefore, the lower mortality rates seen in the lowest income regions may actually represent worse completeness and accuracy of the death registration process.

Given the known difficulties in diagnosing PE clinically^30^ and the low sensitivity of the PE diagnosis in death certificates,^31^ our data probably represent an underestimation of the true mortality rates, a hypothesis strengthened by the much lower PE mortality rates in Brazil when compared to the United States and Europe.^3-8^ The change from ICD 9 to ICD 10 does not seem to have affected our data, since no abrupt changes in mortality rates were observed the year after the conversion in Brazil, which occurred simultaneously in all regions.

## Conclusions

Our study shows a declining trend in PE mortality rates in all age groups over the last 20 years in Brazil, but also documented important differences in these trends amongst the five Brazilian regions and between genders. Although encouraging, the fall in the overall PE death rates was not as marked in women and in the lowest income regions, pointing to possible undesirable disparities in health care access and quality in these groups.
